# Proteomics of hot-wet and cold-dry temperaments proposed in Iranian traditional medicine: a Network-based Study

**DOI:** 10.1038/srep30133

**Published:** 2016-07-25

**Authors:** Hassan Rezadoost, Mehrdad Karimi, Mohieddin Jafari

**Affiliations:** 1 Department of Phytochemistry, Medicinal Plants and Drugs Research Institute, Shahid Beheshti University, Tehran, Iran; 2 Persian Medicine and Pharmacy Research Center, Tehran University of Medical Sciences, Tehran, Iran; 3 School of Traditional Medicine, Tehran University of Medical Sciences, Tehran, Iran; 4 Drug Design and Bioinformatics Unit, Medical Biotechnology Department, Biotechnology Research Center, Pasteur Institute of Iran, Tehran 131694-3551, Iran

## Abstract

Lack of molecular biology evidence has led clinical success of alternative and complementary medicine (CAM) to be marginalized. In turn, a large portion of life Science researchers could not communicate and help to develop therapeutic potential laid in these therapeutic approaches. In this study, we began to quantify descriptive classification theory in one of the CAM branches i.e. Iranian traditional medicine (ITM). Using proteomic tools and network analysis, the expressed proteins and their relationships were studied in mitochondrial lysate isolated from PBMCs from two different temperaments i.e. Hot-wet (HW) and Cold-dry (CD). The 82% of the identified proteins are over- or under-represented in distinct temperaments. Also, our result showed the different protein-protein interaction networks (PPIN) represented in these two temperaments using centrality and module finding analysis. Following the gene ontology and pathway enrichment analysis, we have found enriched biological terms in each group which are in conformity with the physiologically known evidence in ITM. In conclusion, we argued that the network biology which naturally consider life at the system level along with the different omics data will pave the way toward explicit delineation of the CAM activities.

From a mechanistic biology point of view, complementary and alternative medicine (CAM) have long been struggled with a lack of molecular biology evidence. Technological advances accompanying with new multi-disciplinary integrative approaches in biomedical studies help to bridge this gap^1^. It is expected to emerge correlations between molecular and CAM clinical evidence which are functionally connected. Among other fields of CAM, Traditional Chinese medicine (TCM) is the most fortunate to be expressed in the language of modern molecular biology (1–9). Although the new findings based on omics technology, network analysis, and modeling in TCM clarify the similar basis of much traditional ethnic medicines due to humorism as common theory, there are still a lot of unanswered questions in this era.

The common dominator between the systems’ level understanding of biological systems, called systems biology, and the various branches of CAM is the holistic approach or considering the whole system consisting subjects and interactions^1^,^2^. This aforementioned approach could cause to mine emerging properties of biological phenomena. For instance, Ma *et al*. underlined the genetic background of Cold syndrome using the microarray expression profiling and systems biology methods. Their study revealed that genes related to energy metabolism, neurotransmitter, hormones and cytokines are differentially expressed and significantly enriched in the Cold Syndrome based on protein-protein interaction network (PPIN) analysis^1^. Also, Aiping Lu’s research group confirmed Hot and Cold classification in rheumatoid arthritis patients using gene expression and metabolite profiles following by the network analysis methods applied in systems biology. In the underlying biological network, they found the enrichment of G protein signaling pathway, oxidation-reduction in fatty acid metabolism and T cell proliferation pathways in the highly connected clusters of PPIN involved in rheumatoid arthritis patients^3^,^4^,^5^,^6^. There are several other studies which prompted to pave the way for the development of TCM into a new historical period, mainly divided into three categories; TCM-based disease classification^3^,^4^,^6^,^7^,^8^,^9^, TCM network pharmacology^10^,^11^,^12^ and biological investigations on manual therapy such as acupuncture and cupping^13^.

In the Iranian traditional medicine (ITM), the human body categorized into some classes or temperaments based on signs and symptoms similar to TCM^14^,^15^,^16^,^17^. Although several forenamed omics-based and non-omics-based researches have been performed on describing some of these temperaments, there are still many undefined points in the theory of ITM and TCM. In this study, the proteomic profiles of two types of temperaments were compared to each other in order to describe molecular foundation of ITM human body classification theory and candidate molecular biomarkers in these temperaments. It should be noted that in the present study, the people who represent Hot-Wet (HW) and Cold-Dry (CD) temperaments were compared. These categories are two of four combinatory temperaments, produced by two humors; Sanguine and Hypochondria respectively, expressed in ITM, TCM and Ayurveda^11^,^18^,^19^,^20^.

## Results

**Figure 1:**
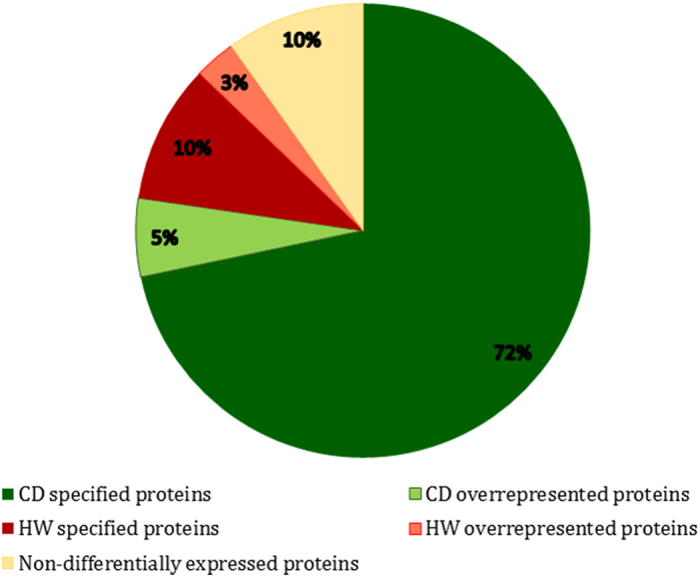
The identified proteins in temperaments. The ratio of the specified and differentially expressed gene products are presented in each temperament i.e. cold-dry and hot-wet.

The identified proteins in HW group in comparison with CD is presented in Fig. 1. Some of the identified proteins are specifically related to each group and some of them are differentially overexpressed in specified group. As it shown in Fig. 1, the most of the differentially identified proteins (72%) are related to the CD group which is indicated the level of complexity in this group. Previously, some DEGs were introduced in similar work focused on Hot and Cold syndrome in TCM conducted by Ma *et al*. and Chen *et al*. in healthy and patient individuals^1^,^3^. They revealed the genetic background of these syndrome using mRNA microarray technology. Although grouping in our study is somewhat different with above-mentioned studies and we analyzed differential expression at the protein levels, some proteins expression are in conformity with their results including FLNB, FLNA, SRC, YWHAZ, UBQLN4, HSPA1A, H2AFX, and MYO5B.

Then, focusing on the pathway enrichment analysis, 17, 6, and 4 pathways are listed based on KEGG, Panther and Reactome databases, respectively. These pathways are significantly enriched in each identified protein set (adjusted p-value < 0.05) are presented in Table 1. Some of the pathways are uniquely disturbed among each group such as dilated cardiomyopathy, Endocytosis, Hypertrophic cardiomyopathy (HCM), Leukocyte trans-endothelial migration, Muscle contraction and Nicotinic acetylcholine receptor signaling pathway in CD individuals. The pathways such as ECM-receptor interaction, Hematopoietic cell lineage and vascular smooth muscle contraction were enriched only in HW individuals.

Conspicuously, these enriched pathways are in harmony with the physiological signs and symptoms stated in the ITM theory. In addition to human body classification including HW, CD, Hot-Dry (HD) and Cold-Wet (CW), the human body organs are also classified using these temperaments which are the main constituent of that organ based on ITM theory^21^,^22^,^23^. For instance, the heart’s temperament is HW and all diseases which functionally impaired toward decreasing cardiac efficiency are diseases related to CD temperament. Therefore, significancy of the enriched terms like Viral Myocarditis, Dilated cardiomyopathy and Hypertrophic cardiomyopathy (HCM) in CD temperaments is in accordance with the biomedical significancy prevalent in ITM. This consistency is occurred in several other cases such as Leukocyte Transendothelial migration and Integrin signaling pathway in CD group which conforms to diverse chronic autoimmune and inflammatory disease are common in people with advanced CD symptoms (CD Syndrome); or Parkinson pathway and Blood coagulation which typically classified in CD signs and symptom. Considering the discussed conformity about CD temperaments, in addition, Hematopoietic cell lineage and Vascular Smooth muscle contraction pathways are significantly enriched in HW temperaments as expected based on ITM explanation for HW sings and symptom.

**Figure 2:**
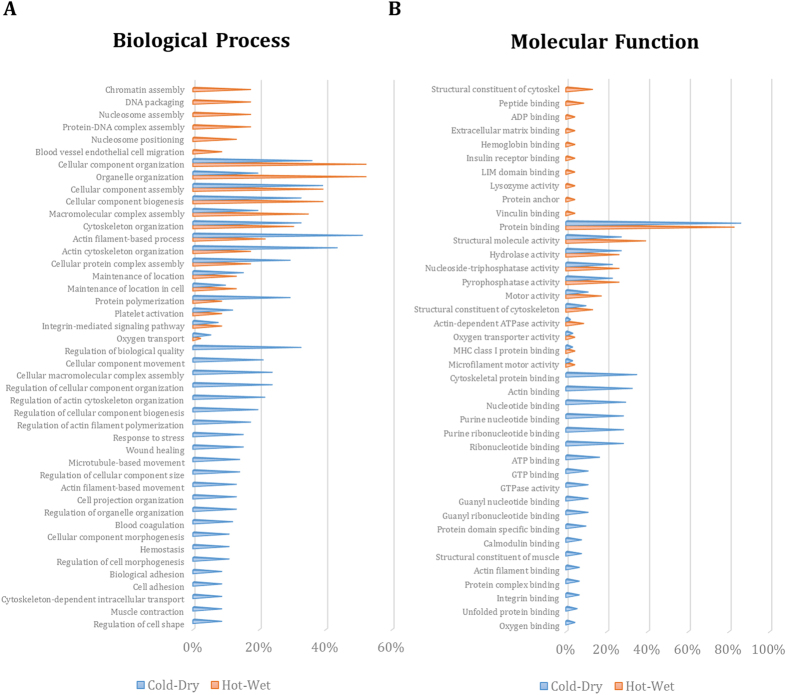
The enriched biological processes (BP) and molecular functions (MF). The statistically significant enriched BP (**A**) and MF (**B**) are listed based on gene ontology database in each temperament. Some of them are presented in both temperaments with different frequencies. The p-values related to each term are listed in Supplementary file 1.

In the next step, the gene ontology related to the identified proteins were explored based on enrichment analysis (Fig. 2). Diverse similar or unique biological processes (BPs) and molecular function (MFs) were distributed among the both groups. The HW specified BPs were generally related to DNA packaging and protein-DNA complex assembly while the CD related BPs were cellular component morphogenesis, movement and transport. The binding to GTP, ATP, Actin and other cytoskeletal constitutes versus the binding to ADP and extracellular matrix is another difference between CD and HW individuals. Interestingly, some of the enriched terms are previously reported in the gene ontology analysis of DEGs found by microarray^3^ including response to stress (GO:0006950), response to wounding (GO:009611), protein localization to cell surface (GO: 0034394), regulation of apoptotic process (GO: 0042981), activation of immune response (GO:0002253), generation of precursor metabolites and energy (GO:0006091), meiotic cell cycle process (GO:1903046), small GTPase mediated signal transduction (GO:0007264), Rho protein signal transduction (GO:0007266), intracellular signal transduction (GO:0035556) and defense response (GO:0006952). The conformity of the gene ontology enrichment analysis is improved in the next step when the PPIN structure is considered to evoke functional associations between differentially expressed proteins.

**Figure 3:**
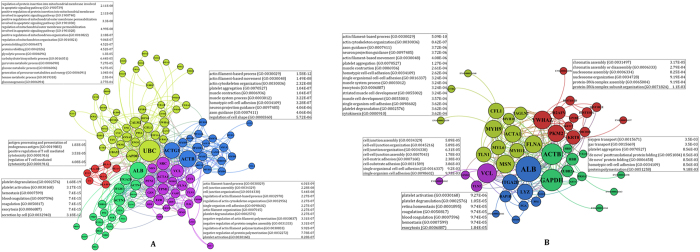
The protein-protein interaction network. The PPIN involved in the cold-dry (**A**) and hot-wet (**B**) temperaments are presented separately. The proteins are sorted by node size based on degree centrality in ascending order and colored differently based on interconnectedness known as graph modules.

To more clarify protein functions, the protein-protein interactions of each specified groups were extracted via STRING database. As it is shown in Fig. 3, the protein-protein interaction network (PPIN) associated with each temperament are represented with sorted node size based on degree centrality. In each PPIN, the modular structures were represented by different color. The high degree proteins in each module are listed in Table 2. The high degree proteins were mostly related to cytoskeletal proteins in both groups. The actin and actin-associated proteins were dominants in the CD while other structural proteins such as tubulin beta and myosin constituted the central nodes in HW PPIN. Interestingly, the pyruvate kinase PKM was overrepresented in HW group while the polyubiquitin-C was overexpressed in CD group. It indicates the role of energy metabolism in HW versus protein degradation in CD individuals along with the complex involvement of cytoskeletal and cell to cell signaling apparatus. The overall BPs in the modules of each network are represented in the figure according to their significant level. The enriched terms related to mitochondria permeabilization and organization accompanying with different processes related to metabolic precursor generation and energy consumption conformed the dissimilar basal metabolism in two studied ITM patterns. Additionally, the repeated processes related to the actin polymerization are also the indication of cytoskeletal organization, cell movement and connection, and signal transduction to discriminate between CD and HW pattern (Fig. 3A). On the other hand, the specified PPIN module related to chromatin assembly bold the importance of gene expression regulation in HW pattern. The gas transport and post-translational folding processes observed in this network discriminate the HW and CD patterns too (Fig. 3B). The proteins related to muscle cell development and contraction, actin filament-based movement and cell-to-cell junction processes also divide the network into distinct modules in HW similar to CD patterns to highlight the importance of signal transduction in developing these patterns.

**Figure 4:**
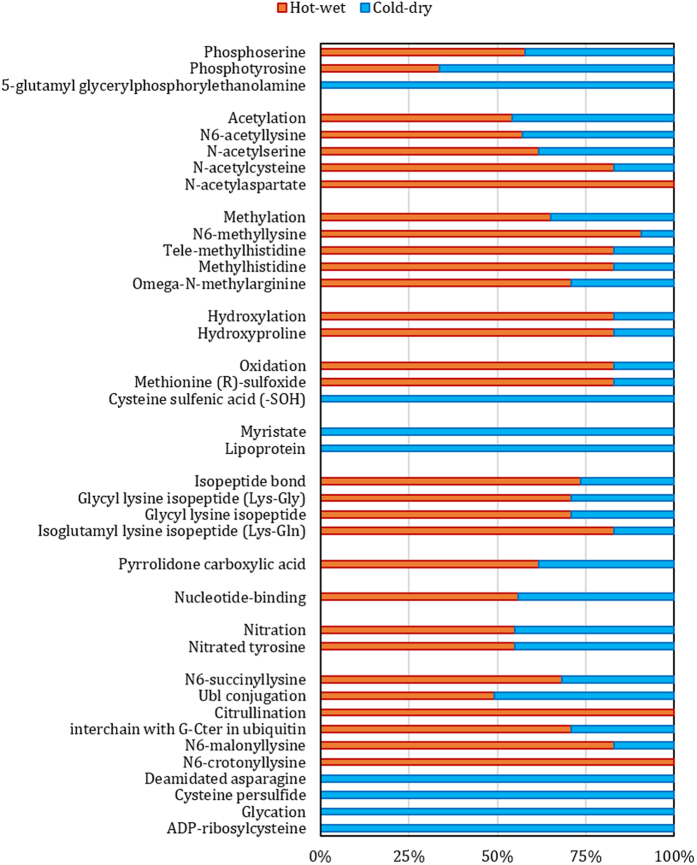
The post-translational modification enrichment analysis. The ratio of the chemical modifications on proteins in both groups are compared together. These terms are statistically significant in enrichment analysis using PEIMAN software.

The activity of proteins is totally affected by different kinds of post-translational modifications situate on protein sequence. These effects could be considered as activation-inactivation or high-low activity which enable signal transduction pathways triggered toward contradictory sides^24^,^25^. In order to discover post-translational modification distribution, the PEIMAN software was used in both groups and compare the results. As it is shown in Fig. 4, the statistically significant enriched PTMs are varied in the many cases. Overally, the various types of phosphorylation, lipoprotein, myristate, Cycteinesulfunic acid, deamidated asparagine, cycteinepersulfide, glycation, and ADP-ribosylcysteine are dominant in CD pattern while in the HW groups other kinds of PTMs are dominantly disturbed such as various types of acetylation, methylation, hydroxylation and citrullination.

## Discussion

The four humors i.e. Sanguine, Hypochondria, Bile and Phlegm, which constitute nine various temperaments or patterns with specified signs and symptom are the key terms in ITM theory. With this classification, the many physiological and pathological events could be categorized and consequently a distinct therapeutic strategy could be identified. The emphasis of these classifications when it emerged that we face to heterogeneous diseases such as rheumatoid arthritis or migraine considering the necessity of personalized medicine. Additional efforts have been projected to elucidate the biomedical foundation of traditional ethnic medicine classification of live phenomena. These efforts include a wide range of basic researches from biothermodynamics to molecular biology investigations^14^,^26^,^27^. In conclusion, it seems that the network biology which naturally consider life at the system level will pave the way toward this goal.

## Material and Methods

### Materials, volunteers and sample preparation procedure

Materials and methods used in this project are reported earlier in detail^16^,^28^. In a nutshell, normal volunteers (aged from 18 to 24 years) were classified in two temperaments according to a questionnaire and physical examination which are commonly used by ITM physicians: Hot-Wet (4 cases), and Cold-Dry (6 cases). The nine indicators were used in order to determine temperaments in ITM; touch, body features, hair features, background color of the body, sleep-wake pattern, waste material (feces, urine and sweat), organ size, temperamental manifestation of warmth, coldness, dryness and moisture on function and behavior, mental states and mood.

Then, peripheral blood mononuclear cells (PBMCs) were extracted from 5 ml of human blood by Ficoll^28^. In the next step, the mitochondrial lysate isolated from PBMCs to extract their related proteins. Detergents and salts were removed by buffer exchange methods, and proteins were solved in 50% acetonitrile and stored at −80 °C. These are subjected to a previously described tryptic digestion protocol^29^,^30^ so that they are reduced, alkylated, and finally subjected to tryptic digestion.

All of the materials and reagents were supplied from Sigma-Aldrich including EDTA, SDS, TEMED, TCA, PMSF, CHAPS, DTT, Bis Acrylamide, Acrylamide and etc. Protein ladder was provided from Fermentase (Page ruler unstained ladder). The central apparatuses used in this study are itemized below: sonicator (Hielscher), refrigerated centrifuge (Kendro, D37520), electrophoresis tools (PayaPajoohesh), speed vacuum (Scanvac), a nano-LC column (obtained from Phoenix S&T, Chester, PA, USA), Easy-nLC system (Thermo Fisher) and a LTQ-Orbitrap mass spectrometry system (Thermo Fisher).

Study procedure was approved by the institutional Ethics Committee of the Pasteur Institute of Iran, and all experiments were performed in accordance with relevant guidelines and regulations issued by the Committee. Informed consent was obtained from all the individuals.

### Proteome analysis

An Easy-nLC system (Thermo Fisher) equipped with a capillary column (150 × 0.075 mm) was used for LC– MS/MS analysis of the tryptic samples. The column obtained from Phoenix S&T (Chester, PA, USA), and the slurry was packed in-house using a 5-μm, 100-Å pore size Magic C18 stationary phase resin (MichromBioResources, Auburn, CA, USA). The chromatography gradient using mobile phase A (0.1% formic acid in deionized water) and the mobile phase B (0.1% formic acid in acetonitrile) was designed for a linear increase from 0 to 8% B in 5 min, 5 to 25% B in 100 min, 25 to 45% B in 10 min, and 45 to 60% B in 10 min. Then an LTQ-Orbitrap mass spectrometry system (Thermo Fisher) was used for identification of peptides from digested proteins. The Xcalibur system (version 2.1; Thermo Fisher) was used to generate peak lists. The optimized parameters were as follow: Orbitrap full MS scans acquired from m/z 350 to 1500 at a resolution of 15 000 (at m/z 400) using an automatic gain control (AGC) value of 2 × 105, the minimum threshold was set to 100 000 ion counts, parent ions fragmented using the LTQ (isolation width of 2 m/z units) with a maximum injection time of 100 ms combined with an AGC value of 1 × 104 using three fragmentation modes such as collision-induced dissociation (CID) alone, electron-transfer dissociation (ETD) alone, and decision tree-based CID/ETD. For ETD MS/MS, the reagent ion source emission current, reagent ion electron energy, and reagent ion source chemical ionization pressure were set to 35 mA, 70 V, and 26 psi, respectively. The activation time and dynamic exclusion time was set to 100 ms and 30 s, respectively. Internal calibration was performed using the background polysiloxane ion signal at m/z 445.120025 as the calibrant. The Agilent 6530 Accurate-Mass Q-TOF combined with the nano chip HPLC system (Agilent, Wilmington, DE, USA) was employed for peptide identification^31^.

### Enrichment and network analysis

Visualization of networks and the global network properties analysis were implemented in Gephi^32^. Given a network, nodes and edges are representative of proteins and pairwise interactions extracted from STRING 10 database^33^. Network clustering was implemented using the fast unfolding clustering algorithm to identify network modules^34^.

Enrichr was used to recognize overrepresented terms in the annotations of HW and CD specified proteins before and after network module finding analysis^35^. This tool was applied to scrutinize whether the protein set were involved in common biological processes (BP), molecular functions (MF), KEGG, Reactome and Panther pathways. These terms in Enrichr output were designated and filtered with adjusted P-value < 0.05. Additionally, Post Translational Modification Enrichment, Integration and Matching Analysis software (PEIMAN 1.0) was used to compare enriched PTMs in two temperaments^36^. Using hypergeometric test, the enriched PTMs were selected considering adjusted P-value < 0.05.

## Additional Information

**How to cite this article**: Rezadoost, H. *et al*. Proteomics of hot-wet and cold-dry temperaments proposed in Iranian traditional medicine: a Network-based Study. *Sci. Rep.* **6**, 30133; doi: 10.1038/srep30133 (2016).

## Supplementary Material

Supplementary Information

## Figures and Tables

**Table 1:** The enriched biological pathways.

Database	Term	Cold-Dry		Hot-Wet	
KEGG		Frequency	P-value	Frequency	P-value
	Antigen processing and presentation	9%	3.68E-03	13%	1.39E-03
	Dilated cardiomyopathy	7%	5.72E-03		
	ECM-receptor interaction			9%	2.08E-02
	Endocytosis	10%	1.38E-03		
	Focal adhesion	17%	1.21E-07	9%	1.26E-02
	Gap junction	10%	1.27E-05	9%	2.20E-02
	Hematopoietic cell lineage			9%	2.70E-02
	Hypertrophic cardiomyopathy (HCM)	7%	4.08E-03		
	Leukocyte transendothelial migration	9%	6.30E-04		
	Malaria			9%	7.87E-03
	Pathogenic *E. coli* infection	13%	6.04E-11		
	Phagosome	14%	6.08E-06		
	Regulation of actin cytoskeleton	17%	3.84E-08		
	Tight junction	15%	6.88E-09		
	Vascular smooth muscle contraction			9%	3.24E-02
	Viral myocarditis	13%	1.22E-08		
**PANTHER**					
	Blood coagulation	8%	1.03E-04		
	Cytoskeletal regulation by Rho GTPase	16%	8.94E-11	17%	9.94E-04
	Hedgehog signaling pathway	5%	2.52E-03		
	Integrin signaling pathway	12%	7.70E-04		
	Nicotinic acetylcholine receptor signaling pathway	9%	1.85E-04		
	Parkinson disease	9%	4.98E-04		
**REACTOME**					
	Hemostasis	24%	2.67E-13	22%	2.13E-03
	Integrin cell surface interactions	7%	4.33E-03		
	Metabolism of proteins	11%	2.06E-03		
	Muscle contraction	4%	8.01E-03		

The statistically significant enriched pathways are listed based on KEGG, Panther and Reactome databases in each temperament. Some of them are presented in both temperaments with different frequencies.

**Table 2:** The important proteins in both groups based on PPIN analysis.

Protein Name	Group	UniProt ID	Description
UBC	CD	P0CG48	Polyubiquitin-C
ACTG1	CD	P63261	Actin, cytoplasmic 2
SRC	CD	P12931	Proto-oncogene tyrosine-protein kinase Src
ACTA1	CD	P68133	Actin, alpha skeletal muscle
ITGA2B	CD	P08514	Integrin alpha-IIb
CFL1	CD	P23528	Cofilin-1
MYH11	CD	P35749	Myosin-11
TLN1	CD	Q9Y490	Talin-1
YWHAZ	HW	D0PNI1	Epididymis luminal protein 4
MSN	HW	P26038	Moesin-1
MYH9	HW	P35579	Myosin-9
PKM2	HW	P14618	Pyruvate kinase PKM
MYL6	HW	P60660	Myosin light polypeptide 6
H2AFV	HW	Q71UI9	Histone H2A.V
TAGLN2	HW	P37802	Transgelin-2
TUBB2A	HW	Q13885	Tubulin beta-2A chain

The CD and HW high degree proteins are listed respectively.
