# Supplementary material for: Proteomics of hot-wet and cold-dry temperaments proposed in Iranian traditional medicine: a Network-based Study
Source: Sci Rep. 2016 Jul 25;6:30133. doi: 10.1038/srep30133 (PMC4959000; doi:10.1038/srep30133)
Supplement: Supplementary Information [file srep30133-s1.docx]

# Proteomics of hot-wet and cold-dry temperaments proposed in Iranian traditional medicine: a Network-based Study

**Hassan Rezadoost^1^, Mehrdad Karimi^2,3**^,Mohieddin Jafari^4*^**

Supplementary file 1:

The enriched biological processes (BP) and molecular functions (MF) with the statistically computed p-value.
